# Advances in basic biology of alfalfa (*Medicago sativa L.*): a comprehensive overview

**DOI:** 10.1093/hr/uhaf081

**Published:** 2025-03-10

**Authors:** Yuanyuan Zhang, Lei Wang

**Affiliations:** State Key Laboratory of Forage Breeding-by-Design and Utilization, Institute of Botany, Chinese Academy of Science, No.20 Nanxincun, Xiangshan, Beijing 100093, China; Key Laboratory of Plant Molecular Physiology, Institute of Botany, Chinese Academy of Sciences, No.20 Nanxincun, Xiangshan, Beijing 100093, China; China National Botanical Garden, No.20 Nanxincun, Xiangshan, Beijing 100093, China; State Key Laboratory of Forage Breeding-by-Design and Utilization, Institute of Botany, Chinese Academy of Science, No.20 Nanxincun, Xiangshan, Beijing 100093, China; Key Laboratory of Plant Molecular Physiology, Institute of Botany, Chinese Academy of Sciences, No.20 Nanxincun, Xiangshan, Beijing 100093, China; China National Botanical Garden, No.20 Nanxincun, Xiangshan, Beijing 100093, China; University of Chinese Academy of Sciences, No.1 Yanqihu East Road, Huairou District, Beijing 101408, China; Academician Workstation of Agricultural High-Tech Industrial Area of the Yellow River Delta, National Center of Technology Innovation for Comprehensive Utilization of Saline-Alkali Land, No. 8 Zhihui Road, Dongying 257300, China

## Abstract

Alfalfa (*Medicago sativa L.*), a perennial legume forage, has been broadly cultivated owing to a variety of favorable characteristics, including comprehensive ecological adaptability, superior nutritive value and palatability, and nitrogen fixation capacity. The productivity traits of alfalfa, specifically its biomass yield and forage quality, are significantly influenced by a series of determinants, including internal developmental factors and external environmental cues. However, the regulatory mechanisms underlying the fundamental biological problems of alfalfa remain elusive. Here, we conducted a comprehensive review focusing on the genomics of alfalfa, advancements in gene-editing technologies, and the identification of genes that control pivotal agronomic characteristics, including biomass formation, nutritional quality, flowering time, and resistance to various stresses. Moreover, a molecular design roadmap for the ‘ideal alfalfa’ has been proposed and the potential of pangenomes, self-incompatibility mechanisms, *de novo* domestication, and intelligent breeding strategies to enhance alfalfa's yield, quality, and resilience were further discussed. This review will provide comprehensive information on the basic biology of alfalfa and offer new insights for the cultivation of ideal alfalfa.

## Introduction

Alfalfa (*Medicago sativa L.*) is perennial legume forage, known as the ‘Queen of forages’, which has been extensively cultivated owing to numerous favorable characteristics, including wide ecological adaptability, superior nutritive value, good palatability, and nitrogen fixation capacity [1–4]. Alfalfa is originated from Persia and was introduced to Greece during the period of Alexander the Great, and then spread across Europe. Drought and winter survival are two issues in the domestication center of alfalfa, which is located in the steppe regions of present-day Iran and Iraq. The term ‘alfalfa’ is originated from the Arabic language, which stands for ‘the best forage’. The alfalfa cultivation was subsequently introduced to North Africa, southern Europe, and Spain. Then, it was shipped to the West Coast of South America, and subsequently to California ~1850. Alfalfa is a prevalent source of forage protein for animals, cultivated in ~80 countries worldwide, predominantly in the USA, Argentina, and China, with a total area spanning >30 million hectares [1, 5, 6], which plays a pivotal role in the fields of animal husbandry.

**Figure 1:**
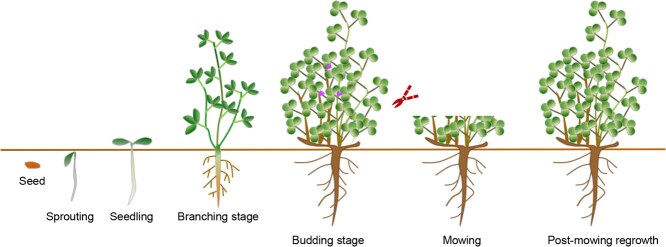
Alfalfa development stages. The growth and development stages of alfalfa include: seed germination, sprouting, seedling (or resprouting) stage, branching stage, budding stage, mowing, and postmowing regrowth stage.

The yield and quality of alfalfa are determined by a variety of factors, including internal developmental factors and external environmental conditions. The growth and development of alfalfa can be categorized into distinct stages: seed germination, sprouting, seedling (or resprouting) stage, branching stage, budding stage, mowing, and postmowing regrowth (Fig. 1). In alfalfa production, farmers usually face a trade-off: early harvesting can boost forage nutrition but hinders regrowth and stand longevity. On the contrary, late harvesting, at full bloom stage, extends stand life and increases biomass yield, but it compromises the nutritive value of the forage [4]. Therefore, flowering time is critical for farmers to determine the optimal time for alfalfa harvesting, thereby maximizing economic benefits.

**Figure 2:**
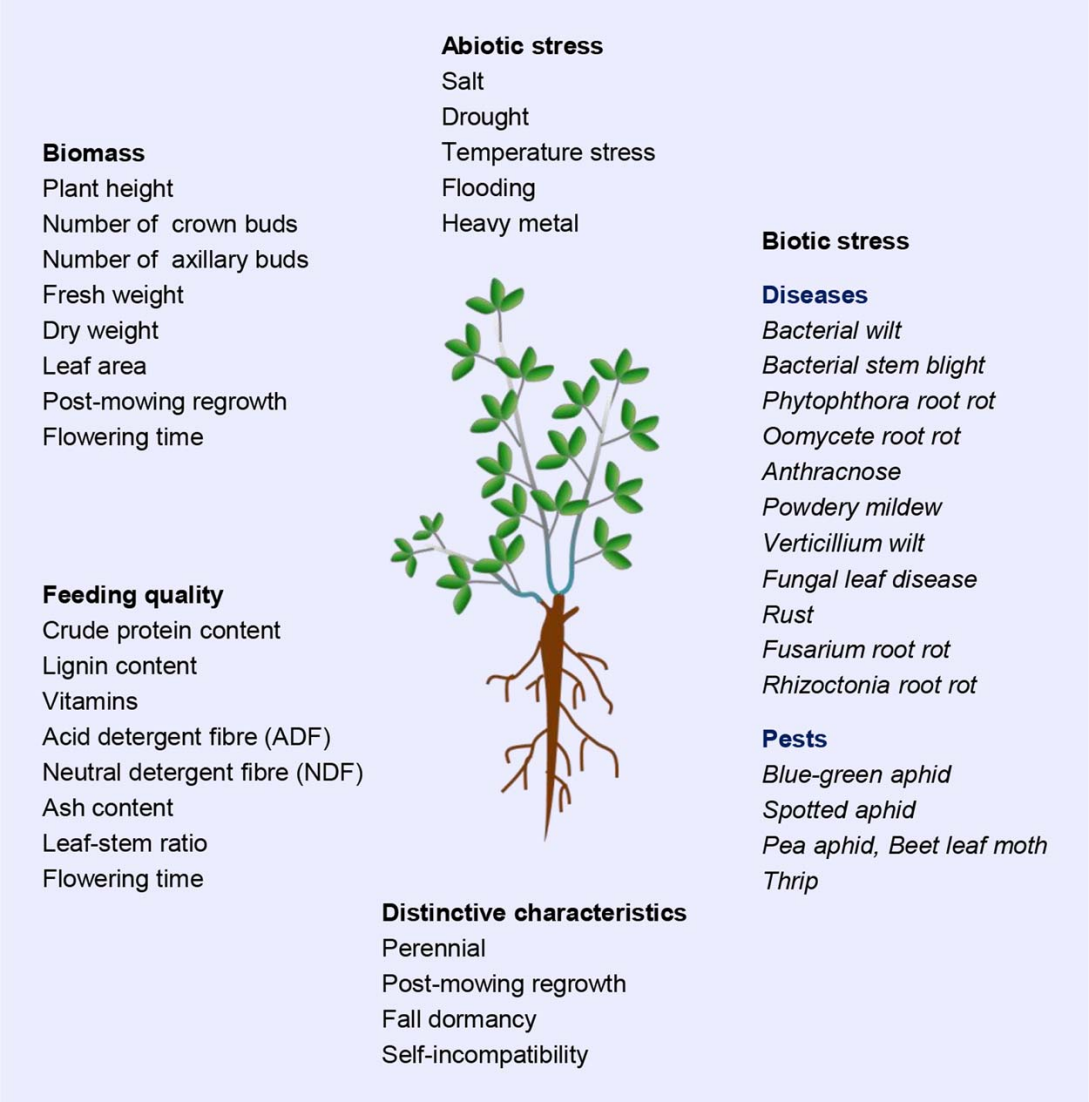
Agronomic traits and distinctive characteristics affecting the yield and quality of alfalfa.

The biomass formation of alfalfa is regulated by various phenotypic factors, including plant architecture, plant height, leaf area, branch number, and other physiological and morphological characteristics. In addition, the yield and quality of alfalfa are also influenced by biotic and abiotic stresses, including salinity, drought, cold, heat, diseases, and insect pests (Fig. 2). Therefore, the improvement of alfalfa yield and quality can be achieved through the regulation of alfalfa flowering time and the cultivation of resilient varieties against a range of biotic and abiotic stresses. Here, we provide a comprehensive overview of the latest advances in the basic biology of alfalfa, which include genome assembly, genome editing, biomass and quality improvement, and the development of stress-resistant varieties. These advances have the potential to significantly enhance the productivity and adaptability of alfalfa. Additionally, we also proposed a molecular design roadmap for an ‘ideal alfalfa’, encompassing more branching numbers, increased plant height and biomass, enhanced nutritive value, and increased resilience to multiple biotic and abiotic stresses, which will provide a framework for the improvement of alfalfa yield and quality. Taken together, this review will provide comprehensive information on the basic biology of alfalfa and offer new insights for the cultivation of ideal alfalfa.

## Genomics of *M. sativa*

Alfalfa is an allotetraploid (2n = 4x = 32) with a large genome (4c = 3.2 Gb), and it is challenging to assemble its genome in the era of high-cost sequencing technology. Lacking high-quality reference genome restrained the molecular biology, genetics, and breeding in alfalfa. However, with the development of third-generation sequencing technology, the genomes of tetraploid and diploid alfalfa were successively deciphered since 2020. An allele-aware chromosome-level genome assembly for the cultivated tetraploid alfalfa ‘XinJiangDaYe’ has been constructed by combining precise single-molecule sequencing with Hi-C data, which consists of 32 allelic chromosomes [1]. Subsequently, a CRISPR/Cas9-based gene editing tool was further developed based on this high-quality reference genome. This system is capable of generating tetra-allelic mutations, leading to significant phenotypic alterations, thus indicating that gene editing system is highly efficient and easy to implement in the cultivated alfalfa, which may facilitate the acceleration of research and molecular breeding in alfalfa [1]. Subsequently, an 816-Mb high-resolution, chromosome-level haploid genome for the autotetraploid alfalfa variety ‘Zhongmu No.1’ has been assembled, with the contig N50 as 3.92 Mb, and in total 49 165 genes were annotated [7]. Further, 137 alfalfa core germplasms and 25 potential ancestral species were resequenced, and genomic population and migration analysis have revealed high genetic diversity and significant gene transfer from wild populations to cultivated alfalfa [7]. Moreover, genome-wide association studies (GWAS) analysis was performed with >30 agronomic traits of 137 alfalfa samples, and discovered >100 potential genomic regions linked to key agricultural characteristics. In addition, it has revealed that the expression of *MsFTa2*, a homolog of *Flowering Locus T*, is upregulated in salt-resistant germplasms, which may affect the dormancy and salt tolerance of alfalfa [7]. Furthermore, an allele-aware chromosome-level genome of autotetraploid cultivated alfalfa ‘Zhongmu-4’ was deciphered [8]. Additionally, a total of 101 single nucleotide polymorphisms (SNPs) associated with 27 distinct agronomic traits were identified via GWAS [8]. Subsequently, two chromosome-scale genomes of diploid alfalfa (*M. sativa* ssp*. caerulea*), an ancestor of autotetraploid alfalfa, were assembled successively [9, 10]. Comparative and evolutionary analysis was performed between *M. sativa* ssp. *caerulea* and *Medicago truncatula* [9]. Numerous structural variations (SVs) were detected between *M. sativa* ssp*. Caerulea* and an autotetraploid alfalfa ‘XinJiangDaYe’ [10]. In summary, three tetraploid cultivated alfalfa genomes and two diploid alfalfa genomes have been assembled (Table 1), which facilitates the molecular genetics and breeding research in alfalfa.

**Table 1:** Information on the three assembled tetraploid cultivated lucerne genomes and two diploid lucerne genomes.

Variety	Ploidy	Species information	Taxon	References
XinJiangDaYe	Allotetraploid	Cultivar	*M. sativa L.*	1
Zhongmu No.1	Allotetraploid	Cultivar	*M. sativa L.*	7
Zhongmu-4	Allotetraploid	Cultivar	*M. sativa L.*	8
PI464715	Diploid	Direct diploid progenitor of autotetraploid alfalfa	*M. sativa* ssp. *caerulea*	9
ZW0012	Diploid	Direct diploid progenitor of autotetraploid alfalfa	*M. sativa* ssp. *caerulea*	10

The potential of gene editing techniques in facilitating precise crop breeding is remarkable. A CRISPR/Cas9-based gene editing technology system has been developed [1]. Further, a CRISPR/Cas9-engineered genic male sterility system was established by targeting the male fertility gene *MsNP1* (*No Pollen 1*) [11]*.* Moreover, an efficient CRISPR_2.0 toolkit, featuring multiple gRNAs, was created for gene editing in ‘Gongnong 1’, a widely grown variety in Northeast China, utilizing an ultrasonic-assisted leaf disc transformation method [12]. An additional CRISPR/Cas9 system has been developed to induce a single nonhomologous end-joining-derived insertion–deletion (indel) at a precise genomic locus in alfalfa [13]. The continuous advancement in CRISPR/Cas9 technology for gene editing in alfalfa promises to revolutionize alfalfa breeding by enabling targeted and efficient genetic modifications, thereby facilitating the development of improved varieties with enhanced traits.

The MODMS (Multi-Omics Database of *M. sativa*) is a comprehensive database that has been established to incorporate data from multiple reference genomes, comparative genomics, high-quality genomic variants, annotations, transcriptomes, proteomics, and metabolomics [14]. The MODMS database is a robust and accessible resource that has a significant impact on alfalfa genomics research.

## Mining of genes regulating critical agronomic traits in alfalfa

The identification of genomic loci or genes regulating various agronomic traits, including forage yield, nutritive value, flowering time, salt tolerance, drought tolerance, temperature tolerance, and biotic stress, is critical for alfalfa breeding. This can be achieved by GWAS in combination with genotypic sequencing (GBS), genome editing, and transgenic technologies.

## Biomass

Alfalfa biomass is a complicated agronomic trait, which is influenced by many phenotypic factors that collectively contribute to its overall development and yield. These factors encompass plant height, plant architecture, branches numbers, leaf area, and several other physiological and morphological characteristics. Plant height is a principal component of plant architecture, exerting a considerable influence on biomass production. Genomic regions related with plant height were identified through a GWAS analysis using 220 accessions of alfalfa worldwide. In total, eight novel SNPs were identified as being substantially associated with plant height. Among them, *Msa0882400*, which is close to one of the SNPs, was annotated as phosphate transporter 3.1, and may serve as a potential candidate [15]. Furthermore, a series of genes that regulate alfalfa biomass have been identified using transgenic techniques. The mutation of *FLOWERING LOCUS Ta1* (*MsFTa1*) through CRISPR/Cas9 editing tools has led to a significant enhancement in forage biomass yield [16]. Overexpression of *stress-induced mitogen-activated protein kinases* (*SIMK*) resulted in a notable enhancement of the aboveground biomass in alfalfa [17]. Melatonin, known as N-acetyl-5-methoxytryptamine, is a multifunctional signaling molecule that is crucial for controlling plant growth, development, and stress responses [18]. N-acetylserotonin methyltransferase (ASMT) is accountable for the final step in melatonin biosynthesis in plants, which is imperative for the synthesis of melatonin [19]. *MsASMT1-*overexpression alfalfa exhibited significantly enhanced biomass, with substantial increases in vegetative growth, plant height, leaf area, stem diameter, cell size, cell number, vascular bundle density, and the number of branches [20]. Overexpression of *MicroRNA156* in transgenic alfalfa plants resulted in a number of significant changes in the plant's characteristics. These included a reduction in internode length and stem thickness, an increase in trichome density and shoot branching, and a delay in flowering time. In addition, there was an elevated level of biomass production, indicating a potential for enhanced agricultural productivity [21]. The *SQUAMOSA PROMOTER-BINDING PROTEIN-LIKE 8* (*SPL8*)-RNAi transgenic alfalfa plants exhibited a significant increase in biomass, reaching up to 43% in the initial harvest and up to 86% in the subsequent harvest [22]. Branching is a pivotal factor in the architecture of plants, with considerable ramifications for the biomass of alfalfa. The plant hormones strigolactones (SLs) have been designated as suppressors of plant branching [23]. The silencing of *MsD14*, which encodes the strigolactone receptor, led to enhanced branching of alfalfa shoots and a significantly increased forage biomass [24]. In summary, alfalfa biomass improvement is a complex process involving the manipulation of multiple genetic and physiological factors. Recent studies have demonstrated the potential efficacy of gene editing, hormone regulation, and signaling molecules in substantially improving plant architecture and yield. However, further investigation is needed to identify additional genes and molecular mechanisms that regulate biomass formation in alfalfa.

## Quality

Nutritive value is another crucial agronomic trait of alfalfa, which directly affects its economic value. Alfalfa leaves are considered to be more nutritious than the stems due to the lignification of the stems, which prevents the digestion of the cell walls. In addition, lignin is regarded as a pivotal factor influencing the nutritive value of forage [3, 25]. Coumarate 3-hydroxylase (C3H) is a member of the cytochrome P450 family of enzymes and plays a pivotal role in the biosynthesis of lignin [26, 27]. The tetra-allelic homozygous *Msc3h* mutant lines created using the multiplex CRISPR/Cas9 system exhibited a marked decrease in lignin content, along with reduced levels of acid detergent fiber (ADF) and neutral detergent fiber (NDF) [28]. Although these alterations did not notably influence biomass yield, they resulted in enhanced digestibility and nutritional characteristics [28]. Comparative transcriptome analyses demonstrate that the NAC transcription factor gene, *NAC SECONDARY WALL THICKENING PROMOTING FACTOR 1* (*MsNST1*), plays a significant role in regulating lignin synthesis in alfalfa [29]. Overexpression of the *HEADLESS* (*HDL*) gene, a homolog of *Arabidopsis WUSCHEL*, induced significant phenotypic improvements in alfalfa. *HDL*-overexpressing plants showed increased branching, greater plant height, and a higher leaf-to-stem ratio, along with enhanced accumulation of fresh and dry biomass. Forage quality assays further revealed elevated levels of crude protein, crude fat, water-soluble sugars, microelements, and both NDFs and ADFs in *HDL-*overexpression plants, collectively enhancing their nutritive value [30]. Vitamin E is a crucial nutrient that animals require from their diets for optimal growth and development. The enzyme γ-tocopherol methyltransferase (γ-TMT), facilitates the conversion of δ- and γ-tocopherols (or tocotrienols) into β- and α-tocopherols (or tocotrienols), playing a pivotal role as the terminal enzyme in the biosynthesis of vitamin E [31]. The content of α-tocopherol was slightly increased in the leaves of *MsTMT-*overexpression transgenic alfalfa plants [32]. Overexpression of *MsTMT* also resulted in delayed leaf senescence [32]. The forage quality was enhanced in the *MsDREB1C-*overexpression alfalfa, exhibiting elevated crude protein and diminished lignin content [33]. The downregulation of *MsFTa1* has been demonstrated to enhance the quality of forage, as evidenced by a notable decline in both NDF and ADF, accompanied by a reduction in lignin content [4]. Taken together, a limited number of genes associated with nutritive value have been identified and additional research is required to uncover more genes that could enhance the quality of alfalfa.

## Flowering time

Flowering time represents a pivotal stage in the growth cycle, marking the transition from the vegetative to the reproductive phase. In contrast to seed-bearing crops, alfalfa is harvested throughout the year for its aboveground biomass. As alfalfa matures, there is a notable decline in the quantity and quality of its nutritional components, particularly in the concentration of nutrients in the leaves, proteins, vitamins, and minerals. Conversely, the proportion of stems, cellulose, and lignification levels increase. To achieve the optimal balance between biomass and feeding quality, the standard practice in alfalfa production is to harvest at the early budding stage. Therefore, it is imperative to elucidate the underlying molecular mechanisms that regulate alfalfa's flowering time. Several genes that regulate flowering time in alfalfa have been identified. Overexpression of *MsSPL20* in alfalfa led to delayed flowering time [34]. CRISPR/Cas9-mediated knockout of the polyester synthase-like genes *MSAD_264347* delayed flowering time in alfalfa (*M. sativa*) [35]. The overexpression of *MsFLAVIN-BINDING, KELCH REPEAT, F BOX 1* (*MsFKF1*), which encodes a putative blue light photoreceptor, has been demonstrated to delay the flowering time of alfalfa plants [36]. The expression of *MsFTa1* is associated with photoperiodic flowering and its downregulation results in a significant delay in flowering time [4, 16]. Overexpression of *MsTERMINAL FLOWERING 1 A* (*MsTFL1A*) delays flowering time in alfalfa [37]. Overexpression of *MsmiR156* in transgenic alfalfa led to a delayed flowering time [21]. Silencing of *MsSPL13* in alfalfa resulted in delayed flowering time [38]. Furthermore, numerous genetic loci and candidate genes linked to flowering time regulation in alfalfa were identified through GWAS, transcriptomic analysis, and quantitative trait loci mapping [39, 40]. Collectively, only a limited number of genes have been identified as being involved in the regulation of flowering time in alfalfa. Further investigation is required to elucidate the molecular regulatory networks that regulate the flowering time of alfalfa.

## Salt

Salinity represents a significant abiotic stress factor influencing alfalfa yield. The development of salt-tolerant alfalfa varieties is critical to improving and exploiting underutilized land, including saline and alkaline land. The identification of genes that regulate this complex agronomic trait will facilitate the development of more effective alfalfa breeding strategies. A number of genes have been identified that are involved in the regulation of salt tolerance in alfalfa. Downregulation of *SPL8* led to significantly enhanced salt tolerance. Conversely, *SPL8-*overexpressing transgenic alfalfa plants were more sensitive to salt stress [22]. Alfalfa transgenic plants overexpressing *MsSPL12* showed improved salt tolerance [41]. By integrating GWAS with transcriptome profiling, eight candidate genes were identified. Among these, five were correlated with resistance to salt stress, while the other three were associated with seed germination under saline conditions [ 42]. Two specific SNPs were identified within the promoter region of the auxin-responsive gene *MsAUX28* that were significantly correlated with salt tolerance [42]. Enhancing the expression of *MsPMTR1*, a putative melatonin receptor, may improve alfalfa growth in saline conditions [43]. The *Defender Against Apoptotic Death* (*DAD*) gene, which was first identified for its role in inhibiting cell death in mammals, plays a pivotal role in the N-glycosylation pathway and acts as an enhancer in the processes of protein folding and the export from the endoplasmic reticulum (ER) [44, 45]. Overexpression of *MsDAD2* enhanced salinity tolerance in alfalfa [46]. The Aquaporin family encompasses a group of membrane proteins that enable the transport of water and certain small molecules through cellular membranes. Nodulin 26-like intrinsic proteins (NIPs) represent a subgroup of the aquaporin family that is unique to plants. Overexpression of *MsNodulin 26-like intrinsic protein 2* (*MsNIP2*) improved salinity tolerance of alfalfa [47]. The *rare cold-inducible 2* (*RCI2*) gene family encodes a highly conserved small molecule hydrophobic peptide. Overexpression of *MsRCI2A*, *MsRCI2B*, *MsRCI2C*, *MsRCI2D*, and *MsRCI2E* could enhance salt tolerance in alfalfa [48, 49]. Overexpression of the *MsWRKY33* gene resulted in improved salt stress tolerance and increased antioxidant capacity, while other agricultural traits remained largely unchanged [50]. Myo-inositol oxygenase (MIOX) serves as a rate-limiting enzyme that irreversibly converts myo-inositol (MI) to D-glucuronic acid (D-GlcA) [51]. The expression of the *MsbZIP53* is significantly induced under saline–alkali stress, which subsequently binds to the promoter region of *MsMIOX2*, thereby activating its expression. The activated *MsMIOX2* protein enhanced alfalfa tolerance to saline–alkali stress by converting MI to D-GlcA, thereby increasing polysaccharide content and participating in the biosynthesis of cell wall pectin and hemicellulose. This strengthens the cell wall and enhances the antioxidant system and photosynthesis [52]. The EMISSION OF BENZENOIDS I/II (EOBI/II), an MYB-type transcription factor, plays a significant role in the floral volatile benzenoid/phenylpropanoid (FVBP) pathway, which is essential for the emission of floral scents [53]. The MsEOBI, an R2R3-MYB transcription factor, has been shown to directly interact with the promoter of *MsPAL1*, a key enzyme in the phenylpropanoid pathway. Overexpression of both *MsEOBI* and *MsPAL1* could enhance the salt stress tolerance of alfalfa, suggesting that the *MsEOBI*-*MsPAL1* module has a great potential in improving salt tolerance of alfalfa [54]. The MsMYB206–MsMYB450–MsHY5 complex modulates alfalfa's salt stress tolerance by regulating flavonoid biosynthesis across diurnal cycles [55]. To conclude, the identification and manipulation of these key genetic factors has demonstrated the potential to significantly enhance alfalfa's tolerance to saline conditions, offering promising genetic targets for the improvement of alfalfa varieties that are better adapted to saline environments.

## Drought

Drought represents a significant threat to alfalfa production. The identification of alfalfa genes that regulate drought response will facilitate the breeding of drought-resistant alfalfa cultivars. *MicroRNA156* (*miR156*) is increasingly being recognized as a potential strategy to improve various plant characteristics. Enhancing the expression of *miR156* in alfalfa leads to improved drought tolerance, which is achieved by the repression of the *SPL13* gene [56]. Alfalfa plants with reduced *SPL9* expression through RNA interference showed improved resistance to drought stress, partly due to the modulation of anthocyanin production pathways [57]. Silencing the *MsSPHK1* gene, which encodes a sphingosine kinase, has been demonstrated to markedly enhance drought tolerance in alfalfa [58]. An MYB-like transcription factor gene (*MsMYBH*) was identified by GWAS of drought resistance in alfalfa. Overexpression of *MsMYBH* substantially improved drought stress tolerance, whereas *MsMYBH*-RNAi plants were more susceptible to drought stress [59]. The plant metalloproteinase *Filamentation temperature-sensitive H* (*FtsH*) encodes an ATP- and Zn^2+^-dependent enzyme that plays crucial roles in responding to environmental stressors. The overexpression of *MsFtsH8* has the potential to enhance the tolerance of PEG-simulated drought stress by stimulating antioxidant capacity [60]. The thiamine thiazole synthase (THI1) plays a pivotal role in regulating thiamine biosynthesis in plants exhibiting a physiological response to diverse abiotic stresses. Transgenic alfalfa plants overexpressing *MsTHI1*, when exposed to drought stress, exhibited higher levels of vitamin B1, soluble proteins, chlorophyll a and b, and increased SPAD values along with heightened peroxidase activity, all of which contributed to improved drought resistance [61]. α-Tocopherol, a key constituent of vitamin E in plants, serves as a powerful antioxidant that neutralizes reactive oxygen species (ROS) generated under photosynthesis and shields lipids from oxidative damage during drought stress. The enzyme γ-Tocopherol methyltransferase (γ-TMT) is pivotal in the biosynthesis of α-tocopherol, catalyzing its formation from γ-tocopherol within the tocopherol biosynthetic pathway. Overexpression of *γ-TMT* increased the accumulation of α-tocopherol in alfalfa [32] and also enhanced PEG-simulated drought tolerance in alfalfa [62]. Dehydrins and aquaporins are essential in the regulation of plant development and stress resilience. Dehydrins offer protective effects, and aquaporins manage the movement of water through cell membranes. The dehydrin MsDHN1 and the aquaporin MsPIP2;1 have been demonstrated to interact with a membrane-bound MYB transcription factor, MsmMYB, in the plasma membrane under nonstressed conditions. Both *MsDHN1* and *MsPIP2;1* positively regulate drought tolerance of alfalfa [63]. In addition, the expression of *MsNUCLEAR TRANSPORT FACTOR 2-LIKE* (*MsNTF2L*) was markedly activated by ABA and drought stress. Elevated *MsNTF2L* levels in alfalfa led to improved resistance to drought, whereas *MsNTF2L*-RNAi transgenic plants demonstrated decreased tolerance to drought, as evidenced by the modulation of leaf water loss in alfalfa [64]. Further, the genomic loci or genes associated with drought resistance traits were determined through GWAS or transcriptomic analysis in alfalfa [65–67]. Taken together, the identification and characterization of these crucial genes involved in the response to drought conditions will facilitate the development of targeted breeding strategies aimed at enhancing alfalfa resilience to drought.

## Heat stress

Heat stress is a key environmental challenge that significantly limits the productivity of alfalfa. Elevated temperatures can disrupt vital physiological functions such as photosynthesis, respiration, and nutrient uptake. These disruptions typically lead to reduced biomass, lower forage quality, and decreased overall yield. Alfalfa plants that have increased *miR156* expression and decreased *SPL13* levels due to RNAi show improved heat stress resistance at 40°C. This is achieved through higher water potential, increased nonenzymatic antioxidant levels, higher anthocyanin content, and greater chlorophyll levels when exposed to elevated temperatures [68]. Additionally, a comparative transcriptome analysis was conducted on plants overexpressing *miR156* under heat stress conditions and identified critical differentially expressed genes (DEGs) and potential targets related to metabolism, stress response, and hormone signaling. These findings highlight the role of the *miR156*/*SPL* network in regulating heat stress tolerance [69]. Moreover, an integrated transcriptomics and metabolomics analysis was conducted on alfalfa leaves exposed to varying high-temperature stresses and revealed significant changes in gene expression and metabolite profiles, particularly within key pathways such as plant signal transduction and glyoxylate metabolism [70]. Collectively, these findings provide valuable insights into the mechanisms underlying high-temperature regulation in alfalfa and highlight the need for further investigation using multilayered approaches.

## Cold stress

As a perennial legume forage, alfalfa's ability to survive winter is crucial for its sustainable development. Thus, understanding the genes and molecular mechanisms underlying its cold stress response is essential for enhancing its tolerance, expanding cultivation areas, and increasing yields. Overexpression of the flavonol synthase gene *MsFLS13* improves the tolerance of alfalfa to combined cold and saline–alkali stress [71, 72]. Additionally, quantitative trait locus mapping and transcriptomic analysis are employed to identify genes or SNPs that are associated with temperature [73–75]. Low temperature also induces fall dormancy in alfalfa, initiating a suite of physiological and biochemical responses that enable the plant to acclimate to winter conditions. These adaptations, including the accumulation of protective compounds and cessation of active growth, are essential for overwintering survival. Thus, elucidating the mechanisms by which cold stress regulates dormancy is critical for optimizing alfalfa management practices and enhancing its winter hardiness. Further investigation is required to identify and characterize the genes that regulate the response of alfalfa to cold stress, which is essential for expanding the range of cultivation conditions for alfalfa and for enhancing yields.

## Biotic stress

Alfalfa faces significant challenges from diseases and pests, which can impact its productivity and quality, leading to substantial economic losses. ‘Bacterial wilt’, ‘Bacterial stem blight’, ‘Phytophthora root rot’, ‘Oomycete root rot’, ‘Anthracnose’, ‘Powdery mildew’, ‘Verticillium wilt’, ‘Fungal leaf disease’, ‘Rust’, ‘Fusarium root rot’, and ‘Rhizoctonia root rot’ are the main diseases of alfalfa [76]. Blue-green aphid, Spotted aphid, Pea aphid, Beet leaf moth, and Thrip are the major pests that threaten the production of alfalfa [76]. An economical and eco-friendly approach to manage pests and diseases is to develop pest- and disease-resistant varieties [76, 77]. Discovering genes that confer resistance to pests and diseases is essential for the breeding of disease-/pest-resistant alfalfa lines. *MsVR38* and *MsVR39*, two linked alfalfa *Verticillium wilt* resistance genes, both are belonging to the TIR-NBS-LRR family [78]. *MsVR39* regulates resistance to *Verticillium wilt* in a positive way, while *MsVR38* regulates it in a negative way [78]. Defoliator insects *Spodoptera litura* larvae are the main threats to alfalfa, which severely affects its yield and quality [79–81]. To determine the function of *miR396* in alfalfa responding to *S. litura* larvae, the *MIM396* was overexpressed into alfalfa. The results showed that sequestering *microRNA396* increased alfalfa resistance, possibly through increased lignin content and biosynthesis of small flavonoids and glucosinolates [82]. To date, only a limited number of genes that confer resistance to diseases and pests have been characterized in alfalfa. It is imperative that future research uncovers additional novel genes to breed varieties with improved resistance to these threats.

## Distinctive characteristics of alfalfa

Perennial, postmowing regrowth and fall dormancy (FD) are distinct characteristics of alfalfa. The perennial and postmowing regrowth nature of alfalfa allows it to regrow multiple times after cutting, thus facilitating repeated harvests throughout the growing season, which are pivotal to its economic significance. Nevertheless, there is a paucity of research investigating the molecular basis of these distinctive characteristics in alfalfa.

FD is an adaptive trait of alfalfa, manifested during the regrowth phase following harvest in late summer or early autumn. FD plays an important role in both biomass production and winter survival at higher latitudes [83]. Nevertheless, the precise physiological, biochemical, and molecular mechanisms that initiate and regulate FD, as well as the genes that are involved, remain largely unclear. Short day (SD) is the primary environmental factor that induces FD in alfalfa. The concentrations of phytochrome B (PhyB) and abscisic acid (ABA) are associated with the photoperiodic reaction and FD in alfalfa [84]. The concentration of leaf PhyB was highest in strongly dormant varieties, moderate in semidormant ones, and lowest in nondormant alfalfa varieties. In accordance with this, the concentration of ABA was also greater in highly dormant varieties [84]. These findings suggest that photoperiod is critical for fall dormancy. However, how photoperiod regulates fall dormancy in alfalfa remains unclear and requires further investigation.

**Figure 3:**
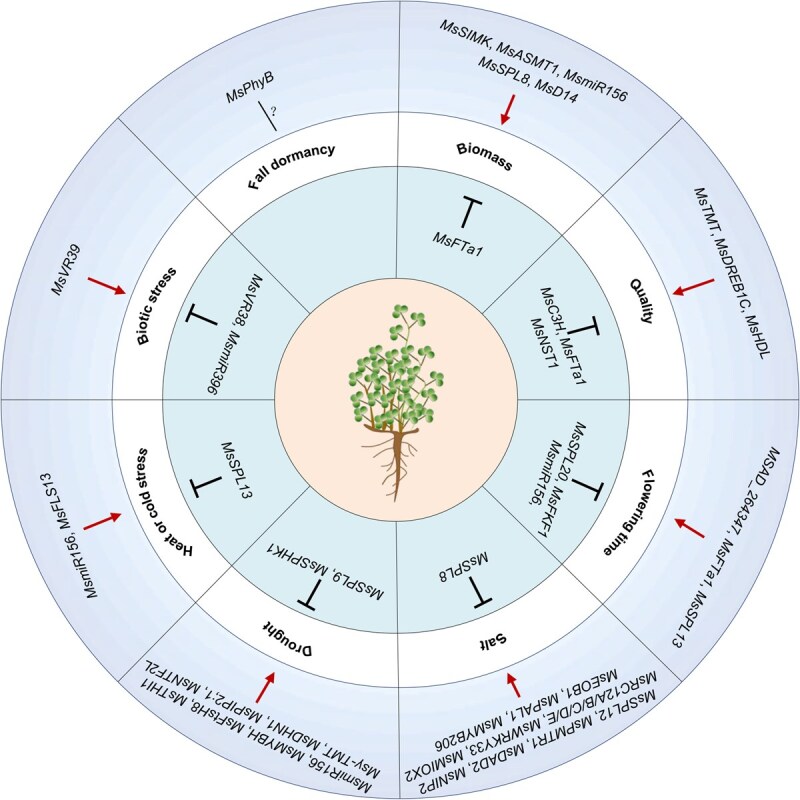
The gene regulatory network for critical agronomic traits in alfalfa.

To summarize, here we reviewed the genes that have been identified in regulating important agronomic traits in alfalfa, including forage yield, nutritive value, flowering time, salt tolerance, drought tolerance, temperature tolerance, biotic stresses, and distinctive characteristics of alfalfa, which will be essential for alfalfa breeding (Table 2; Fig. 3).

**Table 2:** The genes have been used to improve the biomass, quality, and tolerance to biotic and abiotic stresses in lucerne using biotechnology.

Phenotypes	Gene	Positive or negative regulation	References
	*Msa0882400*, phosphate transporter 3.1	/	15
	*FLOWERING LOCUS Ta1* (*MsFTa1*)	Negative	16
	Stress-induced mitogen-activated protein kinases (SIMK)	Positive	17
	*MsASMT1*	Positive	20
	*MsmiR156*	Positive	21
	*SQUAMOSA PROMOTER BINDING PROTEIN-LIKE 8* (*SPL8*)	Negative	22
Biomass	*MsD14*	Negative	24
	*MsCoumarate 3-hydroxylase* (*MsC3H*)	Negative	28
	*MsTMT*	Positive	30
	*MsDREB1C*	Positive	31
	*MsFTa1*	Negative	4
	*MsNST1*	Negative	29
Quality	*MsHDL*	Positive	30
	*MsSPL20*	Negative	34
	*MSAD_264347*	Positive	35
	*MsFKF1*	Negative	36
	*MsFTa1*	Positive	4,16
	*MsmiR156*	Negative	21
Flowering time	*MsSPL13*	Positive	38
	*MsSPL8*	Negative	22
	*MsSPL12*	Positive	41
	*MsAUX28*	/	42
	*MsPMTR1*	Positive	43
	*MsDAD2*	Positive	46
	*MsNodulin 26-like intrinsic protein 2* (*MsNIP2*)	Positive	47
	*MsRCI2A*, *MsRCI2B*, *MsRCI2C*, *MsRCI2D*, and *MsRCI2E*	Positive	48,49
	*MsWRKY33*	Positive	50
	*MsMIOX2*	Positive	52
	*MsEOBI*	Positive	54
	*MsPAL1*	Positive	54
Salt	*MsMYB206*	Positive	55
	*MsmiR156*	Positive	56
	*MsSPL9*	Negative	57
	*MsSPHK1*	Negative	58
	*MsMYBH*	Positive	59
	*MsFtsH8*	Positive	60
	*MsTHI1*	Positive	61
	*Msγ-TMT*	Positive	62
	*MsDHN1*	Positive	63
	*MsPIP2;1*	Positive	63
Drought	*MsNUCLEAR TRANSPORT FACTOR 2-LIKE* (*MsNTF2L*)	Positive	64
	*MsmiR156*	Positive	68
Heat stress	*MsSPL13*	Negative	68
Cold stress	*MsFLS13*	Positive	71.72
	*MsVR39*	Positive	78
	*MsVR38*	Negative	78
Biotic stress	*MsmiR396*	Negative	82
Fall dormancy	*MsPhyB*	/	84

## Future prospects

**Figure 4:**
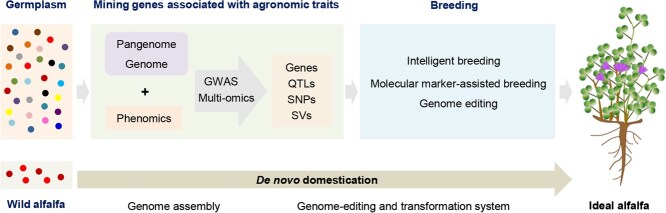
Molecular design roadmap for ‘ideal alfalfa’.

Research conducted in other crop species can offer valuable research paradigms that can be applied to the study of alfalfa. The application of pangenomics, the elucidation of self-incompatibility (SI) mechanisms, the pioneering method of *de novo* domestication, and cutting-edge intelligent breeding techniques represent essential components in the development of a molecular design paradigm for the ‘ideal alfalfa’ (Fig. 4). The ideal alfalfa encompasses more branching numbers, increased plant height and biomass, enhanced nutritive value, and increased resilience to multiple biotic and abiotic stresses, which will provide a framework for the improvement of alfalfa yield and quality.

## Pangenome

An increasing number of studies indicate that it is not possible to capture the full genetic diversity of any species with one or few reference genomes [85–88]. SVs are a main source of genetic variation [89, 90]. However, traditional linear references have challenges with the presentation of genotypes for different alleles at each locus and the identification of larger SVs [88]. The concept of pangenomics and the significance of structural variants are becoming increasingly acknowledged within the plant genomics community [91–94]. Pangenomes have been constructed for a series of major diploid crops, including rice, soybean, maize, pearl millet, sorghum, peas, sunflower, and grapes [88, 90, 95–102]. Polyploid pangenomes have only been constructed in wheat, potato, *Brassica napus*, and cotton [103–106]. Alfalfa is a highly heterozygous, cross-pollinated, and autotetraploid forage crop; the concerns and challenges were discussed and a step-by-step guide to pangenome development for alfalfa has been proposed [91]. Consequently, the development of a pangenome for alfalfa offers great promise for advancing the understanding and enhancement of forage crop. The alfalfa pangenome will facilitate the identification of SVs, which are critical for understanding domestication, environmental adaptation, and phenotypic diversity, also providing insights into core and dispensable genes, enabling the discovery of novel genes and genome-wide patterns of variation associated with important agronomic traits. Taken together, the development of a pangenome for alfalfa is not only a significant step forward in heterozygous and autotetraploid genomics but also a practical tool for enhancing the genetic improvement of this important forage crop.

## Self-incompatibility

SI is a prevalent mechanism in flowering plants that enables plants to recognize and reject self-pollen or pollen from genetically identical individuals, thus promoting outcrossing and preventing inbreeding [107]. Two distinct types of SI have been revealed, namely sporophytic self-incompatibility (SSI) and gametophytic self-incompatibility (GSI). The molecular mechanisms of SI in various plant species were systematically reviewed by a series of preeminent reviews [108–115]. In various plant species, the recognition of self-/nonself SI is governed by a single genetic locus known as the S-locus. Detailed molecular analysis of the S-locus has demonstrated that SI is not a single mechanism but rather a series of divergent mechanisms. In the *Brassicaceae*, these genes encode a pollen ligand and its corresponding stigma receptor kinase, and their interaction triggers incompatible signaling within the stigma's papilla cells. For the Solanaceae-type SI, the key genes are a ribonuclease and an F-box protein, indicating a role for RNA and protein degradation in the process. In the Papaveraceae, the identified female determinant initiates a Ca^2+^-dependent signaling pathway that leads to the rejection of incompatible pollen through cell death [108–115]. Alfalfa is a member of the Fabaceae family and is self-incompatible. Previous studies have identified the S-lineage T2 RNase genes in the genomes of *M. truncatula*, *Trifolium pratense*, *Glycine max*, *Cicer arietinum*, and *Lupinus angustifolius*, but there is no evidence that GSI in Fabaceae is determined by S-RNase lineage genes in Rosaceae, Solanaceae, and Plantaginaceae [116]. A transcriptomic analysis of SI in alfalfa was performed using dissected pistils 24 h after self-pollination and some candidate genes associated with SI were identified [117]. However, the SI in Fabaceae, including alfalfa, is still poorly understood. In summary, while significant progress has been made in understanding the molecular mechanisms of SI across various plant families, the specific mechanisms of SI in Fabaceae, including alfalfa, remain largely elusive and warrant further investigation. SI is a significant barrier to the use of hybrid vigor in alfalfa. Overcoming the mechanisms of SI in alfalfa may lead to more effective use of hybrid vigor to improve both yield and quality.

## Advancing alfalfa breeding with multiomics and cutting-edge tools

Future research on alfalfa should focus on integrating genetic advancements with industrial applications to enhance its resilience and economic value. Developing new alfalfa varieties with increased biomass, enhanced stress tolerance, and improved forage quality is crucial for supporting sustainable livestock production. GWAS offer a promising approach for rapidly identifying genes linked to important traits, thereby accelerating breeding efforts. However, the effectiveness of GWAS can be constrained by population structure and linkage disequilibrium (LD). To overcome these challenges, alternative approaches such as multiomics integration have garnered increasing attention. Integrating GWAS with metabolomics, transcriptomics, and proteomics offers a comprehensive perspective on genetic and biochemical processes, thereby enhancing the identification of candidate genes and their pathways. Moreover, advancements in genomic selection (GS) and machine learning (ML) provide promising alternatives to GWAS. GS utilizes genome-wide marker information to predict breeding values, thereby shortening breeding cycles and enhancing selection efficiency. In addition, ML models, when combined with GWAS, have shown significant improvements in prediction accuracy for complex traits. Taken together, while GWAS is a fundamental tool for rapid gene mining, its integration with other omics approaches and advanced computational methods is essential for maximizing its potential. This integrated strategy will not only enhance alfalfa improvement through precision breeding but also support sustainable agricultural practices.

## *De novo* domestication

Wild species generally have unique advantages such as perennial habits, polyploidy, stress resistance, and natural nutrition. However, there are significant challenges to using wild species in conventional breeding. *De novo* domestication, a novel breeding strategy, has emerged and been demonstrated by pioneer work. Rapid domestication was accomplished in a number of species, such as *Solanum galapagense*, *Solanum pimpinellifolium*, *Physalis pruinosa*, and allotetraploid rice [118–125]. A stepwise strategy for *de novo* domestication has been established in allotetraploid rice: (i) selection of a suitable wild starting material for *de novo* domestication; (ii) assembly and annotation of a high-quality reference genome; (iii) development of an efficient genome-editing and transformation system; (iv) editing multiple genes of domestication and agronomic importance to improve various traits, then certification and popularization of the new crop [123, 126]. This pipeline holds great potential for guiding the domestication of alfalfa, which will provide a new avenue for enhancing yield, quality, and resilience.

## Intelligent breeding

The integration of big data and artificial intelligence (AI) in crop breeding paves the way for a new era of smart breeding [127], significantly improving the efficiency of crop breeding and focusing on forage crops such as alfalfa. This paradigm shift is being driven by rapid advances in gene discovery, biological big data, and AI-based technologies that will revolutionize the approach to crop breeding. AI technologies can analyze large genomic and phenotypic datasets, helping breeders to identify genes correlated with specific traits. AI models, with a particular emphasis on ML and Deep Learning (DL), are increasingly being deployed in the field of crop breeding [128]. In addition, AI enables real-time monitoring of plant growth and health, combined with high-throughput phenotyping, allowing breeders to quickly identify elite varieties in large-scale trials, significantly increasing breeding efficiency [129]. The paradigm of Intelligent Precision-Design Breeding (IPDB), underpinned by AI and big data, targets the optimization of breeding decisions through the synthesis of disparate data sources [127]. The CropGPT initiative can be regarded as an exemplar of IPDB, promoting the dissemination and exchange of breeding expertise via interdisciplinary collaboration and enhancing breeding efficacy [127, 130]. Intelligent breeding promises to revolutionize alfalfa breeding through the application of AI-driven image analysis, which can significantly speed up the selection process and improve the accuracy of phenotypic evaluation, ultimately leading to improved crop productivity and yield.

## Data Availability

All data have been included in the article.
